# Chronic liver disease: Global perspectives and future challenges to delivering quality health care

**DOI:** 10.1371/journal.pone.0243607

**Published:** 2021-01-04

**Authors:** Wai-Kay Seto, M. Susan Mandell

**Affiliations:** 1 Department of Medicine, The University of Hong Kong and The University of Hong Kong-Shenzhen Hospital, Hong Kong, Hong Kong; 2 State Key Laboratory of Liver Research, The University of Hong Kong, Hong Kong, Hong Kong; 3 Department of Anaesthesiology, University of Colorado, Aurora, Colorado, United States of America; 4 National Yang Ming University, Taipei, Taiwan; PLOS, UNITED STATES

## Introduction

Liver disease is a leading cause of mortality worldwide and constitutes a wide range of diseases with varied or unknown aetiologies. For example, in 2017 1.32 million deaths worldwide–or 2 to 4% of all annual deaths–were directly due to cirrhosis [1]. While progress has been made in understanding the causes of liver disease and developing treatments, significant challenges still exist in developing countries and high-income countries alike. In this special collection, we present a curated set of articles reporting new discoveries and advances in epidemiologic, clinical, translational, basic, and psychosocial research from six different continents (Table 1).

**Table 1:** Summary of selected articles highlighting different liver-related epidemiological and clinical issues worldwide.

Article	Country	Disease	Message
Wang S et al. [3]	USA	NAFLD	NAFLD is the leading cause of liver disease and HCC among women receiving liver transplants
Hirose S et al. [4]	Japan	NAFLD	Prognosis in lean NAFLD is similar to obese NAFLD after a median follow-up duration of 19 years
Chen K et al. [5]	Singapore	NAFLD	High rates of advanced liver fibrosis among NAFLD patients with diabetes mellitus
Nielsen S et al. [9]	Denmark	HCV	Linkage to care among drug users essential for HCV elimination
Hanson J et al. [10]	Australia	HBV	Strategies needed in reaching out to Indigenous Australians
Fagundes RN et al. [11]	Brazil	HCV	DAA improved patient-related outcomes, although effects dampened in patients co-infected with HIV
Chiesa A et al. [12]	Uganda	HBV	Residing in internally displaced persons’ camps was a risk factor for HBV in HIV-infected individuals
Lawal MA et al. [13]	Nigeria	HBV, HCV	HBV and HCV prevalence were similar irrespective of HIV infection status

DAA, direct-acting antivirals; HBV, hepatitis B virus; HCV, hepatitis C virus; HDV, hepatitis D virus; HIV, human immunodeficiency virus; HCC, hepatocellular carcinoma; NAFLD, non-alcoholic fatty liver disease.

## Research highlights

The research contained in this collection provides new insights to what is already known in the field and covers a breadth of liver diseases and related challenges.

Several articles included in this collection report research related to non-alcoholic fatty liver disease (NAFLD), which is the most common liver disease worldwide, affecting one-quarter of the population [2]. Wang and colleagues report that liver transplant trends in the United States reflect the emergence of NAFLD as an important cause for end-stage liver disease and hepatocellular carcinoma [3]. However, Hirose and colleagues report a 19-year follow-up study of biopsy-proven NAFLD patients in Japan where the prevalence of diabetes, hypertension, and disease prognosis were similar in lean NAFLD and obese NAFLD, suggesting that risk stratification in NAFLD should therefore not be solely based on one’s body-mass index [4]. Chen and colleagues also report that a high prevalence of advanced liver fibrosis was detected in NAFLD diabetics in Singapore, leading to the question of whether screening for NAFLD or increased liver stiffness measurements should be advocated during diabetes complication screening [5].

Other articles in the collection describe research on viral hepatitis, a major cause of cirrhosis that has a high disease burden throughout the world, with 257 million and 71 million individuals living with chronic hepatitis B virus (HBV) and hepatitis C virus (HCV) infection, respectively [6, 7]. In addition, diagnostic coverage and linkage of susceptible populations to treatment and care have been known to be low globally [8]; indeed, research reported in this collection suggests that these shortcomings still exist. For example, Nielsen and colleagues report that over 50% of HCV positive patients in Denmark had yet to attend specialist care, especially in regions with a likely higher rate of intravenous drug use [9]. In Australia, Hanson and colleagues report that both HBV-related and other liver-related deaths were more common in indigenous Australians when compared to the non-indigenous population [10], suggesting that hurdles still exist in providing quality healthcare to indigenous populations living with HBV in remote areas. On the other hand, treatment coverage for HCV is increasing in many newly developed and developing countries, improving quality of life in those regions. In this collection, Fagundes and colleagues report that subjects in Brazil receiving direct-acting antiviral therapy for HCV achieved significant improvements in health-related quality of life and patient-related outcomes. However, these improvements were dampened in patients with HIV co-infection [11], signifying possible underlying issues related to resource distribution and health care accessibility.

Several papers in this collection also highlight the fact that co-infection with hepatitis viruses and human immunodeficiency virus (HIV) remains a serious public health issue in developing regions, especially in Sub-Saharan Africa. Chiesa and colleagues report that HBsAg prevalence was 7.9% in a population of HIV-infected individuals in Uganda (77% females; mean age 42.8 years), with men having a significantly higher prevalence than women (11.7% *vs* 6.8%). The authors also found that a prior history of residing in an internally displaced persons’ camp was an important risk factor for infection [12], underscoring the need to introduce better preventive interventions to this type of emergency setting. Lawal and colleagues report, however, that among Nigerian children age 2 months to 13 years, the prevalence of HBV and HCV was similar irrespective of HIV infection status, with 5.3% and 4.8% HBV prevalence in HIV-positive and HIV-negative children, respectively, suggesting that HIV-infected children are not more predisposed to viral hepatitis than healthy children [13].

## Conclusion

This collection of papers gives the reader insight into trends of liver disease found across populations, as well as differences inherent to various regions that lead to variation in outcomes. While challenges may differ by region, the management of chronic liver disease will foreseeably continue to take up substantial health care resources at an international scale. Global strategies including the World Health Organization’s aim to eliminate viral hepatitis as a public health threat by 2030 are valuable, but approaches and policies tailored at the local and regional level will also be necessary. Understanding local disease epidemiology, clinical trends, and resulting outcomes will be indispensable in the worldwide battle against liver disease.

## References

[1] Sepanlou SG, Safiri S, Bisignano C, Ikuta KS, Merat S, Saberifiroozi M, et al (2020) The global, regional, and national burden of cirrhosis by cause in 195 countries and territories, 1990–2017: a systematic analysis for the Global Burden of Disease Study 2017. Lancet Gastroenterol Hepatol. 5(3):245–266. 10.1016/S2468-1253(19)30349-8 31981519 PMC7026710

[2] Cotter TG, Rinella M (2020) Nonalcoholic Fatty Liver Disease 2020: The State of the Disease. Gastroenterology 158(7):1851–1864. 10.1053/j.gastro.2020.01.052 32061595

[3] Wang S, Toy M, Hang Pham TT, So S (2020) Causes and trends in liver disease and hepatocellular carcinoma among men and women who received liver transplants in the U.S., 2010–2019. PLoS ONE 15(9): e0239393 10.1371/journal.pone.0239393 32946502 PMC7500679

[4] Hirose S, Matsumoto K, Tatemichi M, Tsuruya K, Anzai K, Arase Y, et al (2020) Nineteen-year prognosis in Japanese patients with biopsy-proven nonalcoholic fatty liver disease: Lean versus overweight patients. PLoS ONE 15(11): e0241770 10.1371/journal.pone.0241770 33186403 PMC7665822

[5] Chen K, Sng WK, Quah JH-M, Liu J, Chong BY, Lee HK, et al (2020) Clinical spectrum of non-alcoholic fatty liver disease in patients with diabetes mellitus. PLoS ONE 15(8): e0236977 10.1371/journal.pone.0236977 32822391 PMC7446805

[6] Blach S, Zeuzem S, Manns M, Altraif I, Duberg A, Muljono DH, et al (2017) Global prevalence and genotype distribution of hepatitis C virus infection in 2015: a modelling study. Lancet Gastroenterol Hepatol. 2(3):161–176. 10.1016/S2468-1253(16)30181-9 28404132

[7] Seto WK, Lo YR, Pawlotsky JM, Yuen MF (2018) Chronic hepatitis B virus infection. Lancet 392(10161):2313–2324. 10.1016/S0140-6736(18)31865-8 30496122

[8] Hutin Y, Nasrullah M, Easterbrook P, Dongmo Nguimfack B, Burrone E, Averhoff F et al (2018) Access to treatment for hepatitis B virus infection–Worldwide, 2016. Am J Transplant, 18: 2595–2598. 10.15585/mmwr.mm6728a2 30025413 PMC6054001

[9] Nielsen S, Hansen JF, Hay G, Cowan S, Jepsen P, Omland LH, et al (2020) Hepatitis C prevalence in Denmark in 2016—An updated estimate using multiple national registers. PLoS ONE 15(9): e0238203 10.1371/journal.pone.0238203 32881877 PMC7470322

[10] Hanson J, Fox M, Anderson A, Fox P, Webster K, Williams C, et al (2020) Chronic hepatitis B in remote, tropical Australia; successes and challenges. PLoS ONE 15(9): e0238719 10.1371/journal.pone.0238719 32881958 PMC7470305

[11] Fagundes RN, Ferreira LEVVdC, Pace FHdL (2020) Health-related quality of life and fatigue in patients with chronic hepatitis C with therapy with direct-acting antivirals agents interferon-free. PLoS ONE 15(8): e0237005 10.1371/journal.pone.0237005 32813740 PMC7437906

[12] Chiesa A, Ochola E, Oreni L, Vassalini P, Rizzardini G, Galli M (2020) Hepatitis B and HIV coinfection in Northern Uganda: Is a decline in HBV prevalence on the horizon? PLoS ONE 15(11): e0242278 10.1371/journal.pone.0242278 33206693 PMC7673526

[13] Lawal MA, Adeniyi OF, Akintan PE, Olorunfemi OS, Temiye EO, Salako AO (2020) Prevalence of and risk factors for hepatitis B and C viral co-infections in HIV infected children in Lagos, Nigeria PLoS One (in press). 10.1371/journal.pone.0243656 PMC7728231 33301507

